# Two Cases in Which the Hair, Teeth and Skin Were Very Imperfectly Developed

**Published:** 1851-04

**Authors:** 


### ARTICLE XIV.

### *Two Cases in which the Hair, Teeth and Skin were very Imperfectly Developed.*

Dr. Thurnam, of York, read before the *Medical and Chirurgical Society*, the cases of two cousins, who were distinguished through life by the almost complete absence of the hair, by the teeth being not more than four in number, by the delicate and defective structure of the skin, and by the absence of sensible perspiration and tears. The author embodied in his paper, a description, by Mr. Erasmus Wilson, of the microscopic character of the skin in one of the cases, from which it appeared that the dermis was in a state of extreme laxity and apparent atrophy. The sudoriparous glands were not wanting, but their ducts were unusually delicate, and their epithelial, and consequently secreting lining, was very thin. The patient died, at the age of fifty-eight, from disease of the lungs. The author pointed out and considered some of the physiological and pathological questions connected with these cases, and concluded his communication with a notice of such analogous instances as have been recorded.

Dr. Williams said the case related in the paper recalled to his mind the case of a young lady who was described to him as being without hair and without teeth. When he saw her, he found that she was not entirely so. She was fifteen years of age; her hair was fine, scanty and white; she had scarcely any hair on her eye-brows, or lashes. She had three or four projections resembling teeth, which were decayed. The nose was imperfectly developed, and she breathed with difficulty through the nostrils. She suffered from chronic ulceration, attended by a discharge, having an unpleasant odor, from the lining membrane of the nares. She had great delicacy of constitution and appearance, and her manner was so timid, that she was thought by some to be imbecile. This, however, was the result of a neglected education. Under proper care she improved. Menstruation was not yet established, although there were indications of its approach. She had never perspired. A tonic plan of treatment had been resorted to.

Dr. Thurnam said, that in the second case detailed in his paper, the patient suffered from ulceration of the nares.

